# Regulation of Neural Stem Cell Proliferation and Differentiation by Graphene-Based Biomaterials

**DOI:** 10.1155/2019/3608386

**Published:** 2019-10-16

**Authors:** Lin Xia, Wenjuan Zhu, Yunfeng Wang, Shuangba He, Renjie Chai

**Affiliations:** ^1^State Key Laboratory of Bioelectronics, MOE Key Laboratory for Developmental Genes and Human Disease, Institute of Life Sciences, Jiangsu Province High-Tech Key Laboratory for Biomedical Research, Southeast University, Nanjing 210096, China; ^2^Zhangjiagang City First People's Hospital, The Affiliated Zhangjiagang Hospital of Suzhou University, Zhangjiagang 215600, China; ^3^ENT Institute and Otorhinolaryngology Department of Affiliated Eye and ENT Hospital, Key Laboratory of Hearing Medicine of NHFPC, Shanghai Engineering Research Centre of Cochlear Implant, State Key Laboratory of Medical Neurobiology, Fudan University, Shanghai 200031, China; ^4^Department of Otolaryngology Head and Neck, Nanjing Tongren Hospital, School of Medicine, Southeast University, Nanjing 211102, China; ^5^Co-Innovation Center of Neuroregeneration, Nantong University, Nantong 226001, China; ^6^Institute for Stem Cell and Regeneration, Chinese Academy of Science, Beijing, China; ^7^Beijing Key Laboratory of Neural Regeneration and Repair, Capital Medical University, Beijing 100069, China

## Abstract

The transplantation of neural stem cells (NSCs) has become an emerging treatment for neural degeneration. A key factor in such treatments is to manipulate NSC behaviors such as proliferation and differentiation, resulting in the eventual regulation of NSC fate. Novel bionanomaterials have shown usefulness in guiding the proliferation and differentiation of NSCs due to the materials' unique morphological and topological properties. Among the nanomaterials, graphene has drawn increasing attention for neural regeneration applications based on the material's excellent physicochemical properties, surface modifications, and biocompatibility. In this review, we summarize recent works on the use of graphene-based biomaterials for regulating NSC behaviors and the potential use of these materials in clinical treatment. We also discuss the limitations of graphene-based nanomaterials for use in clinical practice. Finally, we provide some future prospects for graphene-based biomaterial applications in neural regeneration.

## 1. Introduction

Neural stem cell (NSC) transplantation has become an emerging technology over the past decade for application in tissue engineering and regenerative medicine [1]. More and more studies have focused on all aspects of NSC transplantation, including basic cellular research, transplanted engineering materials, and clinical neural regeneration. Significant progress has been reported in the most recent studies, for example, transplanted neural progenitor cells (NPCs) have been shown to exhibit lineage restriction and site-specific phenotypic differentiation [2–4], fetal brain NPCs have shown promising effects for the improvement of locomotor recovery in spinal cord injury [5–8], and injected human fetal CNS-derived stem cells have been used in clinical trials for the treatment of chronic cervical and thoracic spinal cord injury [9]. These recent works suggest that NSCs are an excellent cellular source for neural regeneration both in scientific research and clinical applications. In addition, NSCs with the potential for self-renewal and pluripotency can monoclonally proliferate and differentiate into neural cell lineages, including neurons, astrocytes, and oligodendrocytes [10]. These two key properties endow NSCs with promising advantages in neural regenerative treatment [1, 11]. NSC fate is determined by several cellular behaviors—including cell adhesion, proliferation, and differentiation—leading to the eventual establishment of cellular functions. Thus, it is necessary to regulate cellular behavior in order to control their final cell fate.

Adhesion, proliferation, and differentiation of NSCs are regulated by numerous growth factors, environmental stimuli, culturing topographies, extracellular matrix (ECM), cell-material interactions, and cell-cell interconnections. Among these factors, cell-material interactions have drawn increasing attention as an important factor for the manipulation of NSC behaviors. The properties of biomaterials such as their topography, stiffness, and composition have been shown to be effective at influencing NSC behavior, mainly through the stimulation of mechanosensors and spatiotemporal dynamics in the cells [12].

With the development of nanotechnology, novel bionanomaterials have become candidates for manipulating NSC behavior both in vivo and vitro. Through nanomanufacturing, the bionanomaterials can precisely simulate the in vivo microenvironment of NSCs and thus regulate their behaviors in the direction of a desired cell fate. As reported in recent studies, porous silicon nanoparticles loaded with growth factors promote the extension of neurites [13], multilayer bacterial cellulose with micropatterns guides the differentiation of NSCs [14], nanofiber scaffolds promote the differentiation of NSCs into neurons [15], and superparamagnetic nanoparticles can track the transplanted stem cells in clinical treatment [16]. In these studies, nanomaterials with variable compositions, dimensions, and topographies could effectively regulate NSC behavior, and the best of these materials have been used in clinical practice.

The novel nanomaterial graphene, a monolayer of sp2-hybridized carbon atoms arranged in a hexagonal honeycomb lattice [17], was first manufactured in 2004 through a method of mechanical exfoliation [18]. Its unique atomic arrangement gives graphene excellent mechanical, electrical, and optical properties [19], and it has been widely applied in biology and biomedicine in the past decade, including biosensing [20], diagnosis [21, 22], drug delivery [23, 24], antibacterial activity [25–27], and cancer treatment [28–33]. Furthermore, because of the common foundational carbon element in both graphene and the human body, graphene possesses good biocompatibility [34–36], which makes it very useful in tissue engineering and regenerative medicine. The family of graphene-based biomaterials has shown promising application in bone regeneration [37], cellular growth scaffolding [38], and surface wound healing [39], and graphene-based biomaterials exhibit significantly enhanced cell attachment, proliferation, and interactions in these applications.

**Figure 1:**
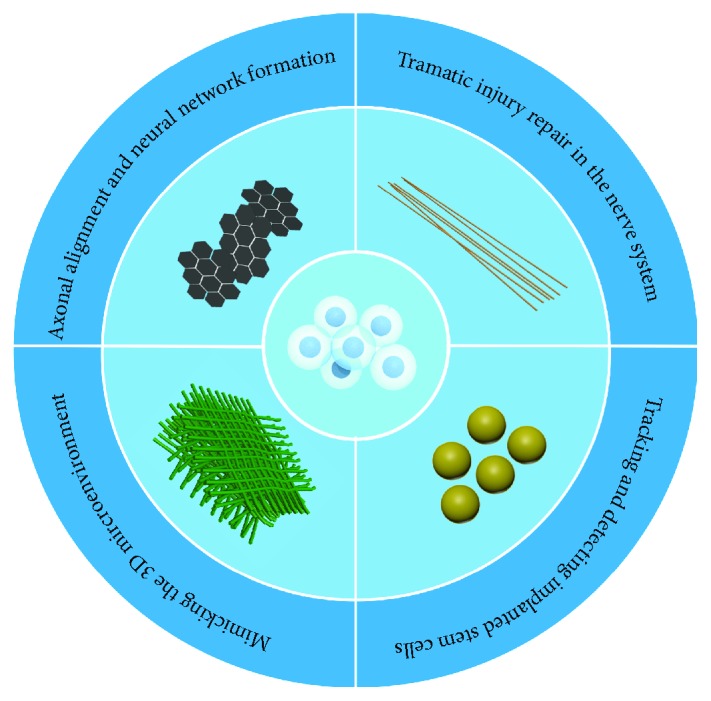
The applications of graphene-based nanomaterials—including particles, 1D fibers, 2D substrates, and 3D scaffolds—in manipulating NSC behaviors.

Based on the development of graphene-based biomaterials and the study of NSC transplantation, novel graphene-based NSCs have emerged as possible clinical treatments for neural degeneration. The integration of graphene-based nanomaterials and NSCs provides a microenvironment that can be manipulated to suit the transplanted cells and can directionally regulate the adhesion, proliferation, and differentiation of the NSCs. Different kinds of graphene-based biomaterials have undergone significant improvements for use in NSC transplantation and have proven promising for use in neural regeneration in clinical trials (Figure 1). In this review, we summarize and discuss recent advances in graphene-based NSC studies, focusing on the type of integration used in the proliferation and differentiation of NSCs, and we discuss expected future applications in clinical neural regeneration.

## 2. Graphene-Based Nanoparticles

**Figure 2:**
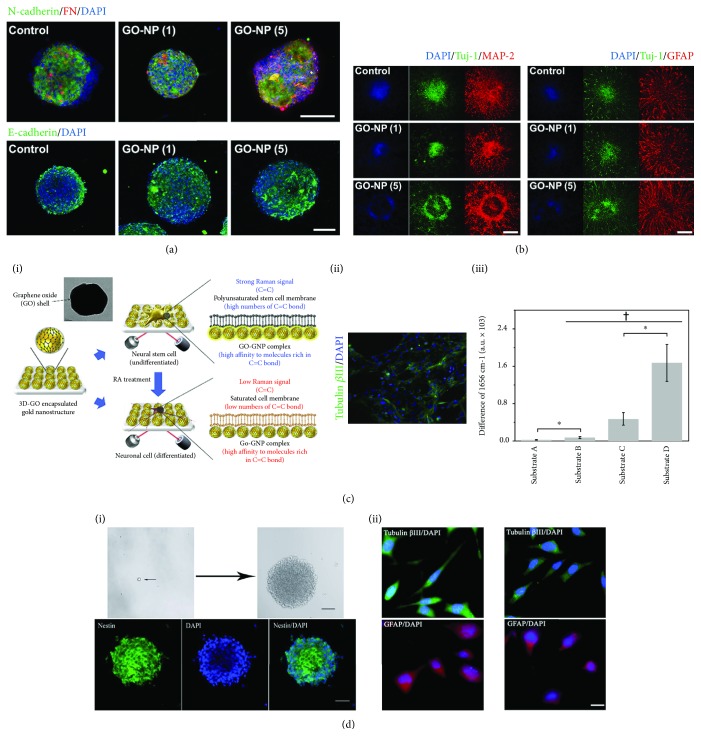
The use of graphene-based nanoparticles to regulate the proliferation and differentiation of NSCs. (a) Immunofluorescence staining of N-cadherin/fibronectin (FN) and E-cadherin in hfNSC neurospheres cultured for 5 days (scale bars = 100 *μ*m). (b) Immunofluorescence staining of differentiated markers (Tuj-1, MAP-2, and GFAP) in hfNSC neurospheres treated for 1 day and for 5 days (scale bars = 200 *μ*m). (c) Graphene-based nanoparticles used for the detection of NSC differentiation: (i) schematic illustration of the detection strategy for NSC differentiation, (ii) differentiation of neural stem cells on the detected substrate, and (iii) the difference in Raman intensities of differentiated and undifferentiated NSCs on different substrates. (d) Graphene quantum dots showed good biocompatibility with the (i) self-renewal and (ii) differentiation of NSCs.

Graphene-based nanoparticles can be internalized by NSCs. Different from the lamellar structure of graphene, the internalized graphene-based nanoparticles can interact with the subcellular organelles, regulate the expression of relevant genes and proteins, and ultimately influence the proliferation and differentiation of NSCs. The study by Kim et al. focused on the self-renewal and differentiation abilities of NSCs after graphene oxide nanoparticle (GO-NP) treatment. Based on immunofluorescence staining, the GO-NP-treated NSC spheres exhibited enhanced cell-cell and cell-matrix interactions, leading to enhanced self-renewal ability. The rate of differentiation of NSC spheres was also accelerated at the same time [40] (Figures 2(a) and 2(b)). In addition to regulating cell behaviors, the graphene-based nanoparticles can also be used to detect NSC differentiation. A novel GO-encapsulated gold nanoparticle was fabricated and used for the detection of the C=C bonds in both differentiated and undifferentiated NSCs. The peak seen in surface-enhanced Raman spectroscopy in the undifferentiated NSCs was 3.5-fold higher than the normal metal structure and was significantly different compared with the differentiated NSCs [41] (Figure 2(c)). Graphene-based nanoparticles were also used to track NSCs in vivo after transplantation, and the fluorescent graphene quantum dots (GQD) fabricated by Shang et al. exhibited low cytotoxicity and highly efficient labeling ability. The immunofluorescence staining indicated that the self-renewal and differentiation abilities were not affected by the GQD, and the GQD showed good biocompatibility and potential use in the bioimaging of transplanted NSCs [42] (Figure 2(d) i, ii). Graphene-based nanoparticles can be internalized into NSCs, and their low cytotoxicity and limited disruption of NSC proliferation and differentiation make them useful for biolabeling and imaging of NSCs after transplantation.

## 3. 1D Graphene-Based Fibers

**Figure 3:**
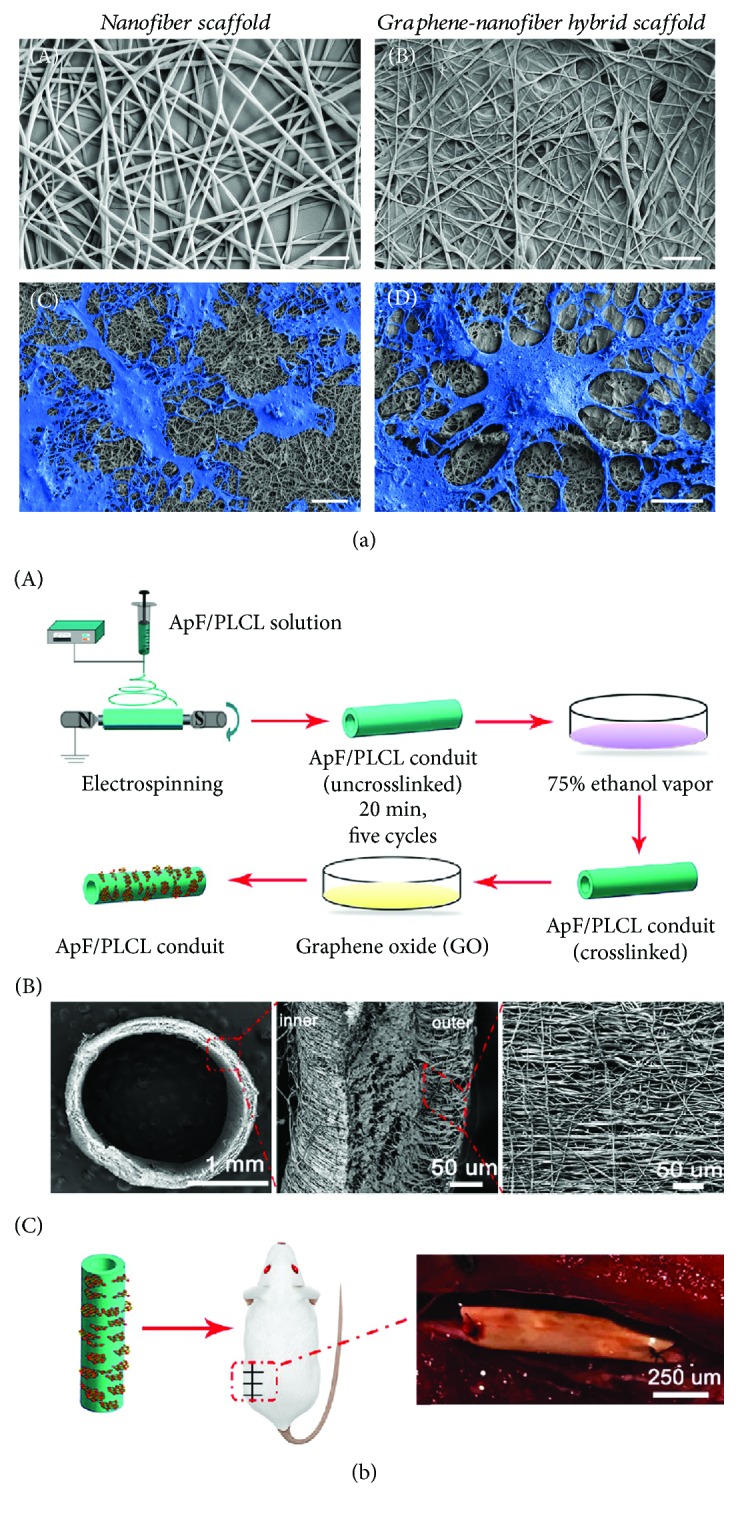
The regulation of the proliferation and differentiation of NSCs by 1D graphene-based nanomaterials. (a) SEM photographs of PCL nanofibers and cultured NSCs. (b) The schematic illustration of the ApF/PLCL conduit (A), the characterization of the ApF/PLCL conduit (B), and sciatic nerve defect repair using an ApF/PLCL conduit (C).

Graphene-based fibers have proven to be very useful in neural injury repair. With the integration of the neural interfacing effect provided by graphene and the “contact-guidance” topography offered by fibers, graphene-based fibers can enhance the neural differentiation of NSCs and direct the oriented extension of neural axons to bridge gaps in injured and severed nerves. The fibers could also be used in the construction of a 3D culturing system for NSCs because of excellent spatial assembly. The fibers could be classified as nano- and microfibers based on diameter size. Nanofibers have a linear morphology within the diameter size range of 1 nm to 100 nm. Nanofibers are usually manufactured from biocompatible materials like PCL (polycaprolactone), PLL (poly-L-lysine), and PLGA (poly(lactic-*co*-glycolic acid)). In recent years, nanofibers manufactured from graphene-hybridized nanomaterials have been heavily studied and used in NSC transplantation. Shah et al. reported that electrospun PCL nanofibers covered with a thin layer of GO could promote NSC differentiation into oligodendrocytes [43] (Figure 3(a)). Wang et al. fabricated *Antheraea pernyi* silk fibroin (ApF)/(poly(L-lactic acid-*co*-caprolactone)) (PLCL) nanofibers by electrospinning, and GO was coated onto the surface of the nanofibers. The Schwann cells cultured on the nanofibers showed enhanced migration, proliferation, and myelination compared to the controls, and the molecules secreted by the cultured Schwann cells also promoted the differentiation and focal adhesion kinase expression of PC12 cells. Animal experiments showed the successful repair of a 10 mm sciatic nerve defect using the nanofibers [44] (Figure 3(b)). In another study, GO sheets were wrapped onto the surface of electrospun nanofibers and then reduced to construct a graphene shell made of hybridized nanofibers. The electrospun nanofiber graphene shell exhibited improved conductivity, mechanical strength, and flexibility, and neurites of primary motor neurons seeded onto these hybridized nanofibers grew much faster than neurites grown on TCPS or graphene film under electrical stimulation of 100 mV. The expression of maturation-related proteins like Tuj-1, MAP-2, and Tau also indicated a higher level of neural differentiation on the hybridized nanofibers compared to TCPS and graphene film. These results indicate that the graphene-shelled nanofibers can strongly promote the growth of primary motor neuron neurites, which has been a long-standing limitation in the repair of damaged central nervous system components, and thus they have great potential for practical use in neural regeneration [45]. Compared with nanofiber, the diameter of microfiber was much larger. Gonzalez-Mayorga et al. fabricated GO microfibers coated with PLL and N-cadherin for NSC implantation, and the newborn neural lineages covered most of the microfiber surface area. These microfibers have also been embedded in hydrogels and implanted at the site of spinal cord injury in rats, and immunofluorescence staining indicated good neural differentiation of NSCs in the perilesional areas, at the material interface, and at the injury site, which suggests the huge potential application of these materials in neural regeneration [46]. In summary, graphene-based fibers, including nanofibers and microfibers, integrate conductivity and implantation properties for enhanced neuronal differentiation, and this along with their good biocompatibility indicates their potential for clinical application in the repair of damaged neurons.

## 4. 2D Graphene-Based Substrates

**Figure 4:**
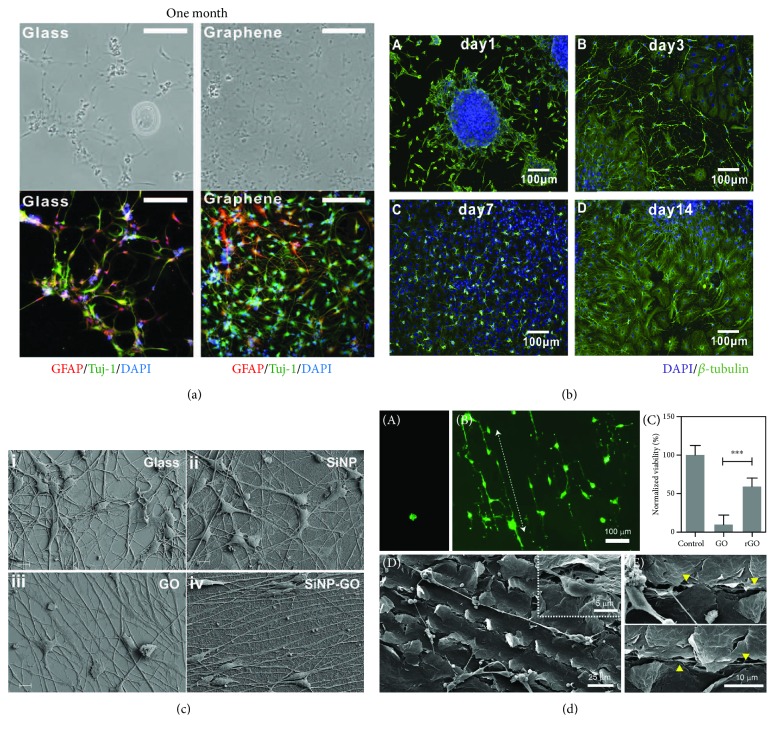
The regulation of NSC differentiation by 2D graphene-based substrates. (a) Graphene substrates enhanced the differentiation of NSCs after culturing for 1 month. (b) Graphene promoted the formation of neural networks after culturing for 14 days. (c) GO-coated SiO_2_ nanoparticles guided the axonal alignment of differentiated NSCs: (i) glass substrate, (ii) SiO_2_ nanoparticle-coated substrate, (iii) GO substrate, and (iv) GO-coated SiO_2_ nanoparticle substrate. (d) Rat primary hippocampal neurons cultured on GO and laser-scribed rGO substrates: (A) primary hippocampal neurons on the GO substrate; (B) primary hippocampal neurons on the laser-scribed rGO substrate; (C) the viability of primary hippocampal neurons cultured on both substrates; (D) a primary neuron cultured on the laser-scribed rGO substrate for 5 days, with the inset image showing a soma attached to a scribed region; (E) neurites passing in between the grooves of the laser-scribed rGO substrate (the arrowheads indicate neurites).

Graphene and GO contain many wrinkles and ripples on their surfaces, and these make them very suitable substrates for NSC adhesion [47]. GO and reduced GO (rGO), as well as other derivatives of graphene, also show high protein adsorption [48] and acceleration of the proliferation and differentiation of stem cells through molecular interactions between the substrate and the cells [49]. Several studies have introduced enhancements to graphene and its derivatives in terms of their effects on the proliferation and differentiation of NSCs. Park et al. found increased adhesion of human NSCs onto the surface of graphene compared to glass at 10 h after seeding, without any significant difference in the number of nestin-positive cells (a marker of pluripotency) between the two surfaces, and human NSCs growing on graphene formed a neural network with greater density compared to the cells growing on glass after differentiation for 1 month [50] (Figure 4(a)). Guo et al.'s study also indicated that NSCs seeded on graphene surfaces adhere and spread onto the surface confluently without any significant difference compared to control cells cultured on TCPS (tissue culture polystyrene substrate). Immunofluorescence staining indicated that most of the seeded NSCs were pluripotent for differentiation into neural lineages [51]. Tang et al.'s study observed the formation of neural networks from seeded neurospheres on the surface of graphene after differentiation for 14 days (Figure 4(b)), and the neural network exhibited obvious changes in cellular Ca^2+^ concentration under electrical stimulation [52]. These studies all demonstrate the effect of graphene and its derivatives on enhancing NSC proliferation and differentiation while maintaining all of the NSCs' neural cell properties.

Graphene-based composite nanomaterials have become an emerging area of focus with the development of modern nanomanufacturing methods. Through hybridization with biocompatible and biodegradable materials, graphene-based composite nanomaterials integrate electrical, cytofriendly, and biodegradation properties, providing potential nanomaterials for clinical use in neural regeneration. Solanki et al. introduced a hybrid nanomaterial manufactured by coating 300 nm SiO_2_ nanoparticles onto the surface of a GO sheet. The NSCs seeded onto the hybrid material adhered and spread onto the surface after 24 h, and the cells began to align after differentiating for 2 days. The neurites began to outgrow and became aligned after differentiating for 5 days. On the final day after 2 weeks of differentiation, the differentiated cells seeded on the surface of the graphene-SiO_2_ hybrid material exhibited good alignment and extended axons [53] (Figure 4(c)). Akhavan and Ghaderi fabricated graphene nanogrids on an SiO_2_ substrate containing TiO_2_ nanoparticles (GONR grid) and seeded NSCs onto the surface of the hybrid material. Immunofluorescence staining for nestin indicated that the NSCs on the GONR grid exhibited enhanced attachment and proliferation compared to quartz, TiO_2_-nanoparticle/SiO_2_, and rGONR grid substrates, and the NSCs preferred to align along the grid patterns. After flash photostimulation, there were significantly more neurons on the rGONR grid and TiO_2_-NPs/SiO_2_ substrates compared to the samples that were kept in the dark, and the neurons preferred to form two-dimensional neural networks around the grid patterns [54]. Lee et al. used laser scribing to generate rGO patterns on GO film, and the scribed patterns offered physical contact guidance cues with conductivity for the attached neurons. Rat primary neurons attached and spread onto the laser-scribed GO substrate, and the neurons elongated along the laser-scribed patterns. The somas of the neurons made contact with the scribed region, and the majority of the neurites were within the scribed grooves [55] (Figure 4(d)). Yang et al. developed a GO-based culture substrate with hierarchical microgrooves of different widths. The NSCs seeded on the substrates expressed significantly greater amounts of integrin and underwent differentiation more readily compared to controls. In addition, the alignment of the neuronal cytoskeleton improved as the microgrooves became thinner [56]. The graphene-based hybrids in the above studies all enhance the alignment of differentiated cells and direct the extension of neurites, and this facilitates the anisotropy of the neural pathway and suggests that these materials are promising approaches for clinical applications in neural regeneration.

## 5. 3D Graphene-Based Culturing Systems

Although the 2D graphene-based substrates offer a facilitated culturing environment for NSCs that can regulate cell behaviors such as proliferation and differentiation, successfully mimicking the 3D ECM microenvironment in vivo has been a remaining obstacle for tissue engineering and regenerative medical applications. The in vivo 3D ECM is composed of growth factors and physical topographical cues from ECM proteins and fibers, and the microenvironment formed by the ECM supports and regulates the proliferation and differentiation of NSCs. In comparison to 2D culturing substrates, 3D culturing systems are more facilitative to the culturing of NSCs since they (1) maintain the cells' natural morphology in vivo, (2) enhance proliferation depending on the specific materials and cell types, (3) direct differentiation under the guidance of specific materials and topographies, and (4) upregulate the expression of differentiation-relevant genes and proteins. Thus, it is necessary to set up in vitro 3D culturing systems for simulating the ECM microenvironment of NSCs. During the process of developing 3D culturing systems for NSCs, graphene-based biomaterials have been studied and developed further. Several new kinds of novel three-dimensional graphene-based nanomaterials for the proliferation and differentiation of NSCs have been reported in the past decade, including porous materials, fibers, and scaffolds.

**Figure 5:**
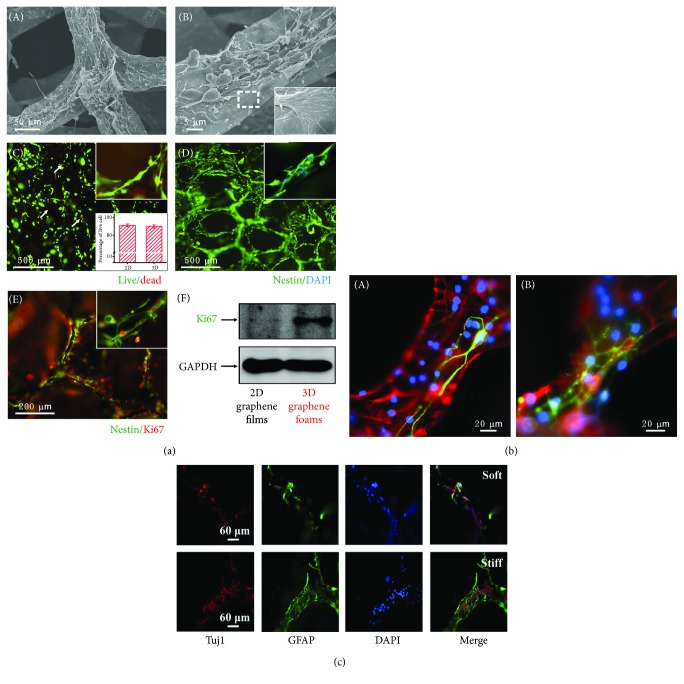
Regulation of the proliferation and differentiation of NSCs on 3D graphene-based nanomaterials. (a) The proliferation of NSCs cultured on 3D graphene foam: (A) low-magnification SEM images of NSCs cultured on 3D graphene foams; (B) high-magnification SEM images of NSCs cultured on 3D graphene foam; (C) cell viability assay of NSCs cultured on 3D graphene foam; (D) immunofluorescence staining for nestin in NSCs cultured on 3D graphene foam after culturing for 5 days; (E) immunofluorescence staining of Ki67 and nestin in NSCs cultured on 3D graphene foam; (F) western blot analysis of Ki67 expression on 2D graphene films and 3D graphene foams. (b) The differentiation of NSCs on 3D graphene foam. (c) The differentiation of NSCs on stiff and soft graphene foam scaffolds.

Graphene foam is constructed as a porous 3D structure and has been proven in the past few years to be an excellent 3D culturing system for NSCs in tissue engineering applications. Graphene foam can effectively enhance NSC adhesion, proliferation, differentiation, and neural network formation [57] in vitro, and the differentiated cell lineages grown in such culturing system exhibit good directionality. The expression of oligodendrocyte markers is much lower than the expression of neuron and glial cell markers, which indicates the directed differentiation into neuronal lineages when NSCs are grown on graphene foam [58] (Figures 5(a) and 5(b)). Besides, the graphene foam also integrated the 3D culturing environment with the excellent electrical characteristics of graphene that could offer the 3D culturing environment under electrical stimuli for NSCs. This integration was very facilitative to the study of NSC behaviors under electrical stimuli both in vivo and vitro. However, poor biodegradability is still a major challenge to the clinical application of graphene foam, and the development of novel biodegradable composite graphene foam substrates is high on the agenda for future studies in neural regeneration.

Graphene-based scaffolds are another key approach to mimicking the NSC microenvironment for clinical applications in neural regeneration. Different from the 3D spatially aligned graphene-based fibers, scaffolds show more complicated 3D structures, including fibers, surfaces, and interconnections. Thus, such scaffolds are more similar to the in vivo ECM microenvironment of NSCs compared to other materials. In addition to more precisely simulating the microenvironment of NSCs, the scaffolds also integrate physiological cues in the 3D geometry like surface wrinkles and ripples of graphene and the contact-guidance of fibers. NSCs seeded in the 3D geometry of graphene-based scaffolds receive regulatory signals from the surrounding environment, and this allows their behaviors to more closely mimic those of cells in vivo. Using nanomanufacturing technology, regulatory cues can be integrated into the scaffolds during the manufacturing process in order to improve the neuronal differentiation of NSCs.

A study by Ma et al. established two kinds of 3D scaffolds made of graphene foams with stiff and soft properties, respectively. The stiff scaffold significantly enhanced the attachment and proliferation of NSCs, and the expression of proliferation markers, including BrdU and Ki67, was much higher on the stiff scaffold compared to the soft scaffold. However, the NSCs cultured on the stiff scaffold mainly differentiated into astrocytes. The expression of the neuron marker Tuj-1 was significantly lower than that on the soft scaffold, and the expression of the axonal marker GAP-43 was much higher on the stiff than the soft scaffold. Thus, the stiff scaffold enhanced axon genesis and suppressed the differentiation of neurons [59] (Figure 5(c)). Further to the goal of developing implantable materials, hybridized scaffolds of graphene and medical-friendly materials have emerged and have been applied in trials of neural regeneration. The study by Qian et al. reported the fabrication of a polydopamine (PDA) and arginylglycylaspartic acid- (RGD-) coated graphene-loaded PCL nanoscaffold. The scaffold was composed of three tubular layers, including an inner PDA/RGD layer, an intergraphene (single-layer (SG) or multilayer (MG))/PCL layer, and an outer PDA/RGD layer. All three tubular layers were porous with a diameter of 50 *μ*m to ensure sufficient water and oxygen exchange between the external environment and the tubular scaffold lumen. The immunofluorescence staining indicated that the expression of Tuj-1 was highest in differentiated cells on the PDA/RGD-SG/PCL scaffold, and western blotting also showed increased expression of neurotrophic factors like nerve growth factor on a PDA/RGD-SG/PCL scaffold compared to control scaffolds. After implantation, the walking track analysis indicated that the recovery of the sciatic nerve was faster on the PDA/RGD-SG/PCL and PDA/RGD-MG/PCL scaffolds than on other materials at 6 and 12 weeks after the surgery and was similar to the autograft group at 18 weeks after the surgery [60]. So to summarize, graphene-based scaffolds can more accurately mimic the in vivo NSC microenvironment, and they facilitate NSC attachment, proliferation, and differentiation in a similar manner to the in vivo environment. With the progress that has been made in material modification and nanoconstruction, graphene-based scaffolds will find increased applications in neural regeneration.

## 6. The Future of Clinical Use

Over the past decade, graphene-based materials have shown significant advantages for the guided manipulation of NSC fate by directly regulating the attachment, proliferation, and differentiation of NSCs. Novel graphene-based nanomaterials with the potential for clinical use have been tested in trials for further application in the treatment of neural regeneration. However, for long-term implantation, the mechanisms behind the biodistribution, biocompatibility, and biodegradation of graphene-based materials must be better understood [61].

Similar to the general properties of all nanomaterials, the biocompatibility of graphene-based nanomaterials depends on the morphology, size, and surface modifications of the material. The morphology and size of graphene-based materials are key factors for their uptake by NSCs. Flat GO sheets with a thickness of hundreds of micrometers have been shown to be an excellent neural interfacing substrate for NSC culture [52]. In contrast, graphene nanosheets have been shown to disrupt cellular redox equilibrium resulting from hydrophobic interactions between the nanosheets and the cell membrane [62]. These results emphasize the importance that morphology plays in the biocompatibility of nanomaterials. Dextran-modified GO nanosheets can be cleared without toxicity at 7 days after injection [63], showing that surface modification is another key factor for biocompatibility. Thus, the toxicology of graphene-based nanomaterials can be effectively decreased through the design and modification of the materials' surfaces.

The biodistribution of graphene-based nanomaterials is mainly due to nanoparticles or sheets originating from the implanted biomaterials. The biological effect of distributed nanoparticle materials has been studied systematically, and the introduction of radioisotope tracking has led to significant advances in the study of biodistribution. It has been shown that ^125^I-labeled PEGylated graphene nanosheets accumulate in the reticuloendothelial system (RES), especially in the spleen and liver, after the injection of the material and that the accumulated graphene nanosheets are metabolized through excretion [64]. However, pure GO nanomaterials do not show RES accumulation after intraperitoneal injection, and neither PEGylated nor pure GO cause significant toxicity due to long-term retention in mice. These results indicate that the in vivo distribution of graphene-based nanomaterials depends on the route of administration and the surface modifications of the material.

For long-term in vivo implantation, biodegradation is an important factor to consider regarding clinical treatment. Combined with the metabolization routes, the hybridization of graphene-based and biodegradable nanomaterials offers an ideal strategy. Guo et al. reported a composite material fabricated by assembling rGO nanosheets onto the surface of a 3D bioactive porcine acellular dermal matrix. The hybridized materials showed good enhancement of mesenchymal stem cell differentiation and newborn neurite extension, while at the same time exhibiting good biodegradation [65]. The composite electrospun nanofibers of GO and PLGA also showed excellent degradation properties after being applied in regenerative medicine [66]. Biodegradation can also be controlled by surface modification, and it has been shown that degradation initiated by horseradish peroxidase-induced oxidation can be prevented by PEGylation of the GO surface. Thus, it can be concluded that hybridized graphene nanomaterials based on surface modification have potential use as biodegradable implantation materials for neural regeneration.

## 7. Conclusions

The study of graphene-based nanomaterials for the application in neural regeneration is an emerging area and has shown the potential for such materials in clinical use. Graphene-based nanomaterials combine biocompatibility with the excellent mechanical, electrical, and optical properties of graphene for manipulating NSC fate. However, clinical applications are still at an early stage, and there are still a few issues that need to be addressed, such as the simulation of the in vivo NSC microenvironment, the toxicity of long-term implantation, and the degradation of implanted materials. Significant progress has been made in the use of hybridized nanomaterials and surface-modified graphene, and we anticipate that implanted graphene-NSC systems will provide a solid foundation for further studies of clinical neural regeneration.
